# Increased mortality among HIV infected patients with cryptococcal antigenemia in Guinea-Bissau

**DOI:** 10.11604/pamj.2018.29.18.14099

**Published:** 2018-01-09

**Authors:** Ditte Thomsen, Cecilie Juul Hviid, Bo Langhoff Hønge, Candida Medina, David Da Silva Té, Faustino Gomes Correira, Lars Østergaard, Christian Erikstrup, Christian Wejse, Alex Lund Laursen, Sanne Jespersen

**Affiliations:** 1 Bandim Health Project, Indepth Network, Bissau, Guinea-Bissau; 2 Department of Clinical Immunology, Aarhus University Hospital, Aarhus, Denmark; 3 Department of Infectious Diseases, Aarhus University Hospital, Aarhus, Denmark; 4 National HIV Programme, Ministry of Health, Bissau, Guinea-Bissau; 5 GloHAU, Center for Global Health, School of Public Health, Aarhus University, Aarhus, Denmark

**Keywords:** Cryptococcal antigenemia, cryptococcal meningitis, HIV, antigen screening, lateral flow assay, Guinea-Bissau

## Abstract

Cryptococcal antigenemia may precede development of cryptococcal meningitis and death among patients with advanced HIV infection. Among 200 retrospectively and randomly selected ART-naïve patients with CD4 counts < 100 cells/μl from Guinea-Bissau, 20 (10%) had a positive cryptococcal antigen test. Self-reported headache and fever were predictors of a positive test, while cryptococcal antigenemia was a strong predictor of death within the first year of follow-up, MRR 2.22 (95% CI: 1.15-4.30). Screening for cryptococcal antigenemia should be implemented for patients with advanced HIV in Guinea-Bissau. Pre-emptive anti-fungal therapy should be initiated prior to ART-initiation if the screening is positive.

## Introduction

Cryptococcal meningitis (CM) is a severe opportunistic infection among HIV-infected patients and is estimated to cause almost 625.000 deaths each year globally. The highest prevalence is found in sub-Saharan Africa, where 13-44% of HIV/AIDS-related deaths are due to CM [1]. Cryptococcal antigen (CrAg) is detectable in blood several weeks before meningitis symptoms [2] and thereby precedes the development of CM. The prevalence of cryptococcal antigenemia among HIV-infected patients has been described in several African and Southeast Asian countries, with a prevalence ranging from 1-19%, the highest prevalence found among patients with low CD4 counts [3]. Data from West Africa are sparse. One study has described the prevalence of cryptococcal antigenemia among a smaller group of HIV-infected individuals in Guinea-Bissau and found a prevalence of 1% among patients with a median CD4 count of 185 cells/μl [4]. A high prevalence of cryptococcal antigenemia could be expected in Guinea-Bissau, since late presentation is common [5]. The WHO recommends the use of CrAg screening in antiretroviral therapy (ART)-naïve patients with a CD4 count < 100 cells/μl in populations with a high prevalence of cryptococcal antigenemia (prevalence > 3%). If the CrAg test is positive, pre-emptive anti-fungal therapy prior to ART is recommended. The aim of this study was to investigate the prevalence of cryptococcal antigenemia, risk factors for antigenemia and examine cryptococcal antigenemia as a risk factor for mortality among patients with advanced HIV-infection in Guinea-Bissau, to provide data to guide decision on CrAg screening.

## Methods

**Setting and study design:** All data, except CrAg status, were prospectively collected from patients enrolled in the Bissau HIV Cohort, located at the HIV clinic at Hospital Nacional Simão Mendes, Bissau, Guinea-Bissau [5]. CrAg was measured retrospectively in stored samples. Patients testing HIV-positive at the clinic are invited to be enrolled in the cohort. ART-naïve patients are recorded as lost to follow-up (LTFU) when they do not report to the clinic for 7 months, whereas patients on ART are recorded as LTFU when they are more than 3 months late for their last scheduled visit [5]. Patients are registered as dead if reported by family members or a contact person or confirmed by telephone calls from the clinic. Whenever a CD4 count is performed, surplus plasma is stored in a biorepository in Aarhus, Denmark. Retrospectively, we randomly selected 200 ART-naïve patients with a CD4 count < 100 cells/μl and a corresponding available plasma sample.

**Laboratory analysis:** HIV screening was conducted with a rapid test (Determine HIV-1/2 assay, Abbott Laboratories, Abbott Park, IL, USA) and confirmation and discrimination were performed using the SD Bioline HIV 1/2 3.0 rapid test (Standard Diagnostics Inc, 145 Kyonggi-do, South Korea). During 2012, the SD Bioline HIV 1/2 3.0 was gradually replaced by the First Response HIV card 1-2.0 (PMC Medical, Mumbai, India). CD4 counts were measured by flow cytometry (CyFlowSL, Partec, Munster, Germany). CrAg was measured in stored plasma samples using a semi-quantitative lateral flow assay (CrAg Lateral Flow Assay, IMMY, Inc., Norman, USA) and positive samples were titrated to find the dilutions (between 1:5-1:5120), to which the samples were still positive.

**Statistics:** Baseline characteristics of patients with and without cryptococcal antigenemia were compared using χ^2^-test for proportions and Wilcoxon rank-sum test for continuous data (non-normal distribution). We estimated the time from sample collection until death as well as risk of being LTFU within one year using Cox proportional hazard models. Patients who experienced an event after one year from baseline or were still retained-in-care were right censored at one year. P-value < 0.05 was considered statistically significant. All statistical analyses were carried out using Stata IC 13.0 (StataCorp, College Station, TX, USA).

**Ethical approval:** Prior to enrolment, all patients in the Bissau HIV Cohort provided signed and dated informed consent or a fingerprint if they were unable to read or sign. The study was approved by the National Ethics Committee of Guinea-Bissau 185 (Parecer NCP/No.15/2007).

## Results

**Baseline characteristics and factors associated with cryptococcal antigenemia:** Among 200 randomly selected ART-naïve patients with a CD4 count < 100 cells/μl, 20 (10%) had a positive CrAg test with a median titer of 1:400 (range 1:5-1:5120). Eighty-four men (42%) and 116 women (58%) were included in the study. Median age was 35 (IQR 28-43) years and median CD4 cell count was 21 (IQR 11-38) cells/μl. Among the 200 patients, 162 were HIV-1-infected, 24 were HIV-2-infected and 14 were HIV-1/2 dually infected. There were no statistically significant differences in socio-demographics or clinical presentation, except self-reported headache and feeling feverish, between CrAg positive and negative patients (Table 1).

**Table 1:** Baseline characteristics of study population according to cryptococcal antigenemia status

Variable	CrAg negative	CrAg positive	p-value
N	180	20	
**Socio-demographics**			
Age, years -median (IQR)	35 (28-43)	33.5 (28-39)	0.65
Male sex - n (%)	78 (43.3)	6 (30.0)	0.25
Schooling^1^, yes – n (%)	104 (64.2)	11 (64.7)	0.97
**Clinical presentation**			
CD4 cells/μl- median (IQR)	22 (12-43)	16 (9-28)	0.14
BMI^2^ - median (IQR)	17.6 (15.5-20.2)	17.8 (16.3-20.2)	0.85
Headache^3^, yes- n (%)	116 (64.8)	18 (90.0)	0.02
Fever^4^, yes - n (%)	110 (61.8)	17 (85.0)	0.04
**HIV type**			0.28
HIV-1-n (%)	148 (82.2)	14 (70.0)	
HIV-2 -n (%)	21 (11.7)	3 (15.0)	
HIV-1/2 -n (%)	11 (6.1)	3 (15.0)	
**Co-infections**			
TB treatment^5^, yes -n (%)	19 (10.6)	2 (10)	0.93

**Figure 1:**
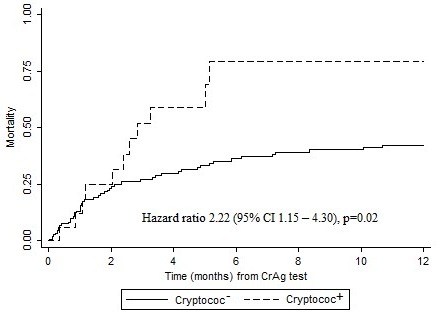
Survival estimates according to cryptococcal antigen status within 1-year of follow-up

**Mortality:** After one year of follow-up, 11 (55%) of the patients with cryptococcal antigenemia had died compared with 51 (28%) of the patients without cryptococcal antigenemia (p=0.01). Cryptoccal antigenemia was a strong predictor of death among CrAg positive patients with a mortality rate ratio (MRR) of 2.22 (95% CI: 1.15-4.30) and an even stronger predictor of death among patients with a CrAg titer diluted more than 5 times (> 1:5), MRR 2.48 (95% CI: 1.28-4.80). After adjusting for CD4 counts, cryptococcal antigenemia remained a strong predictor of death, MRR 2.08 (1.07-4.03). Most deaths occurred within the first few months after CrAg screening (Figure 1). We found no difference in death between men and women (p=0.29) and adjusting for sex did not change the risk of death by cryptococcal antigenemia, MRR 2.23 (95% CI: 1.15-4.31).

**Loss to follow-up:** Within one year, seven (35%) of the CrAg positive patients and 76 (42%) of the CrAg negative patients became LTFU (p = 0.53). Combined endpoint of death or loss to follow-up showed no difference between CrAg positive and negative patients, hazard rate ratio 1.34 (95% CI: 0.82-2.20).

## Discussion

In these 200 patients with CD4 counts < 100 cells/μl, 10% had a positive plasma CrAg test. CrAg positive patients had a higher mortality compared with CrAg negative patients within one year from CrAg detection. The prevalence found in our study is comparable to the prevalence found in the few previous studies from West Africa [4,6]. Except self-reported headache and fever, we found no difference in socio-demographics or clinical presentation between CrAg positive and negative patients. Previous studies have found low CD4 counts, high WHO clinical stage, headache and fever to be associated with cryptococcal antigenemia [2,7]. Including only patients with CD4 counts below 100 cells/μl may be the reason we did not see the same associations between CD4 counts and cryptococcal antigenemia in this study. Presence of plasma CrAg was associated with higher risk of death in our study. Similar associations have been found in previous studies from Sub-Saharan Africa. In Tanzania, cryptococcal antigenemia was an independent predictor of death or being LTFU among ART-naïve HIV-infected adults [8] and in South Africa, cryptococcal antigenemia was an independent predictor of mortality among ART-naïve patients [9]. A study from Kenya found no significant associations between CrAg positivity and increased risk of death [10].

The difference in mortality outcomes between these studies may be affected by the level of CD4 cell counts, clinical status and ART status of the patients. This is the first study in Guinea-Bissau to investigate the prevalence of cryptococcal antigenemia among a group of patients to whom WHO recommends CrAg screening if the prevalence is higher than 3%. Furthermore, it is one of few studies including both HIV-1, HIV-2 and HIV-1/2 infected patients. This study has several limitations. Loss to follow-up was a major challenge in both groups and might have underestimated the amount of deaths in this population. CrAg screening was performed retrospectively on stored plasma samples, symptoms of meningitis were not registered in the database and lumbar puncture was not performed, which means that verification of development of CM among CrAg positive patients was not possible. Information about fluconazole use had not been registered and the use of fluconazole for other purposes might have prevented development of meningitis and death in CrAg positive patients. It is possible that our study either underestimates or overestimates the consequences of cryptococcal antigenemia in Guinea-Bissau. The prevalence of CrAg of 10% and the strong association with death found in this study highlights the importance of screening HIV-infected patients with CD4 counts < 100 cells/μl for cryptococcal antigenemia in Guinea-Bissau. At the moment, recommendation of CrAg screening is not mentioned in the national guidelines; National Programme for The Fight Against AIDS in Guinea-Bissau. Screening patients in high risk of developing CM provides an opportunity to treat these patients with pre-emptive anti-fungal therapy prior to ART and thereby decrease the risk of death by CM, as recommended by WHO. CrAg screening using CrAg LFA is a simple test with high sensitivity and specificity and can be a major advance in meningitis diagnostics in resource-limited settings.

## Conclusion

In conclusion, cryptococcal antigenemia was prevalent among patients with advanced HIV-infection in Guinea-Bissau and a strong predictor of death. Death by CM can, in many cases, be prevented by CrAg screening and pre-emptive fluconazole treatment, why CrAg screening should be considered implemented among HIV-infected patients with CD4 counts < 100 cells/μl in Guinea-Bissau.

### What is known about this topic

- Cryptococcal antigenemia may precede development of cryptococcal meningitis and death among patients with advanced HIV infection;
- The WHO recommends the use of CrAg screening in HIV infected patients with a CD4 count < 100 cells/μl in populations with prevalence of cryptococcal antigenemia > 3%;
- Pre-emptive anti-fungal therapy should be initiated prior to ART-initiation if the screening is positive.

### What this study adds

- The prevalence of cryptococcal antigenemia among guinean HIV-infected patients with a CD4 count < 100 cells/ul were 10%;
- Cryptococcal antigenemia was a strong predictor of death among patients with advanced HIV-infection in Guinea-Bissau;
- CrAg screening should be considered implemented among HIV-infected patients with CD4 counts < 100 cells/μl in Guinea-Bissau.

## Competing interests

The authors declare no competing interests.
